# Data on growth, uptake and N_2_ fixation of grass-clover leys fertilized with mineral N fertilizer and cattle slurry

**DOI:** 10.1016/j.dib.2022.107998

**Published:** 2022-03-02

**Authors:** Doline Fontaine, Rebekka Kjeldgaard Kristensen, Jim Rasmussen, Jørgen Eriksen

**Affiliations:** Department of Agroecology, Aarhus University, Blichers Allé 20, Tjele 8830, Denmark; Present address: Yara GmbH & Co. KG, Hanninghof 35, 48249 Dülmen, Germany

**Keywords:** White clover, Red clover, Ryegrass, Festulolium, Symbiotic N fixation, Herbage composition

## Abstract

This article presents the data obtained from a field experiment in which grass–clover leys were fertilized with increasing N rates applied in either mineral N fertilizer and/or cattle slurry forms. The leys were composed of a 2–species mixture of white clover (*Trifolium repens L*.) and ryegrass (*Lolium perenne L*.) and a 4–species mixture of white clover, red clover (*Trifolium pratense L*.), festulolium (*Festulolium braunii*) and ryegrass. In total, eighty fields were established at two farm sites in the western part of Denmark on sandy soils and monitored for two herbage seasons (2018–2019). Dry matter yield, botanical composition, N concentration and the proportion of N derived from the atmosphere using the 15 N dilution method were recorded in the harvestable biomass after each cut. Furthermore, the specific growth, N uptake and quantitative biological N fixation of the species were determined.

The dataset can be used to establish the N balance, to calculate the optimal economic fertilization rate based on grass-clover composition and to predict N leaching and residual effect.

The data presented were thoroughly used and discussed in the research article “Contrasting effects of slurry and mineral fertilizer on N_2_ fixation in grass-clover mixtures”.

## Specifications Table

**Table:** 

Subject	Agronomy and Crop Science
Specific subject area	N fertilization of grass–clover leys
Type of data	Tables, Excel workbook
How data were acquired	Field experiments performed during two consecutive years at two farmer sites in Western Jutland (Denmark).
Data format	Raw data
Parameters for data collection	Two temporary grassland mixtures, a 2–species and a 4–species leys, were established at two different sites. Ten treatments of N fertilization split throughout the herbage season, including different levels from 0 to 480 kg N ha^−1^ either as mineral fertilizer or combined with a basic application of cattle slurry (120 kg N ha^−1^). The same treatments were re-applied in the second year. The proportion of N derived from the atmosphere (%Ndfa) of clovers was estimated using the 15 N isotope dilution method. The treatments were laid out in a randomized plot design with four replicates.
Description of data collection	Biomass was harvested and sampled five times during the herbage season. Analysis included dry matter yields, botanical composition, N concentration and %Ndfa.
Data source location	South-western Jutland (SW) and Mid-western Jutland (MW); Denmark SW coordinates: 55°32′N, 8°29′E MW coordinates: 56°10′N, 8°46′E
Data accessibility	With the article
Related research article	Kristensen, R.K., Fontaine, D., Rasmussen, J., Eriksen, J., 2022. Contrasting effects of slurry and mineral fertilizer on N2-fixation in grass-clover mixtures. Eur. J. Agron. 133, 126431. https://doi.org/10.1016/j.eja.2021.126431

## Value of the Data

• The dataset provides measurements of the harvestable biomasses for each cut to assess the effect of N fertilization rates and types on clover–grass dynamics. The data were obtained from fields set up at farmer sites which are more representative of real–farm conditions than experimental plots.
• The dataset is of interest to researchers, agricultural advisors and modelers of agricultural systems.
• The dataset can be used to establish the N balance, to calculate the optimal economic fertilization rate based on grass-clover composition and to predict N leaching and residual effect.

## 1 Data Description

The parameters of the experiment are presented in tables and the data obtained are presented in an excel workbook Table 1. presents the main characteristics of the soils at both sites. Mineral N (NO_3—_N and NH_4—_N) contents were taken in spring in both years prior starting of the fertilization treatments Table 2. shows the cumulative precipitation, manual irrigation and mean–air temperature at both sites and years.

**Table 1:** Selected characteristics of the soils at SW and MW sites.

		SW		MW	
		2018	2019	2018	2019
Texture		Sand		Sandy loam	
Clay†	g kg^−1^	67		43	
Silt†	g kg^−1^	59		42	
Fine sand†	g kg^−1^	470		344	
Coarse sand†	g kg^−1^	377		521	
Organic matter†	g kg^−1^	27		50	
NH_4—_N‡	ppm	1.9	1.9	3.5	1.9
NO_3—_N‡	ppm	2.1	0.6	1.9	1.2

† 0–25 cm depth.

‡ 0–75 cm depth.

**Table 2:** Cumulative monthly precipitation, manual irrigation and mean air temperature at both sites in 2018 and 2019.

	Precipitation + irrigation (mm)				Temperature (°C)			
	SW		MW		SW		MW	
Month	2018	2019	2018	2019	2018	2019	2018	2019
January	105.6	56.1	101.8	55.4	2.5	2.5	2.1	2.0
February	26.7	50.4	33.9	63.0	−0.2	4.4	−0.7	4.4
March	31.8	119.6	33.0	137.9	1.0	5.9	0.5	5.4
April	61 + 40	15.9	68+50	10.6	8.7	8.6	8.8	8.3
May	16.2	36.3	20.3 + 50	49.9 + 25	15.5	10.0	15.3	9.4
June	28.5 + 80	42.8 + 30	26.3 + 75	68.4 + 25	16.1	16.1	15.9	15.5
July	36.1	68.1	26.2	99.0	18.3	16.5	18.3	16.2
August	141.8 + 40	71.2	120.8	124.3	17.4	17.7	16.7	16.8
September	135.5	167.3	143.2	170.1	14.3	13.9	13.7	12.8
October	44.5	190.1	47.2	165.5	10.8	10.0	10.3	9.0
November	42.1	130.8	40.1	106.8	6.0	5.9	5.8	5.4
December	94.4	86.7	89.2	103.2	4.7	5.5	4.5	4.8

Table 3 summarizes the composition of the cattle slurry taken from the host farmer storage before the 1st and 2nd cuts, at both sites and years. The quantity of slurry applied was adjusted according to its ammonium-N content to give an annual application of 120 kg plant available N ha^−1^. There are no pH data available in 2019 at MW site.

**Table 3:** Characteristics of the cattle slurry applied before the 1st and 2nd cuts at both sites in 2018 and 2019 [1].

			DM	Total N	Ammonium–N	P	K	
					
Location	Year	Application prior cut	%	kg Mg^−1^				pH
SW	2018	1st	7.8	3.8	2.1	0.6	3.2	5.9
		2nd	6.1	3.4	1.8	0.4	2.6	6.6
	2019	1st	8.0	4	2.2	0.6	3.1	5.6
		2nd	8.0	3.9	2.1	0.4	2.1	5.6
MW	2018	1st	3.0	2.3	1.6	0.3	2.2	6.8
		2nd	7.4	4.5	2.3	0.7	3.7	7
	2019	1st	5.4	3.5	2.0	0.5	2.7	–
		2nd	5.8	3.5	1.9	0.4	2.5	–

Table 4 represents the distribution of the different fertilizer types and rates applied during the herbage season in both years and sites. Note that the same treatment was given during the two consecutive years.

**Table 4:** Nitrogen application treatments distribution during the growth season in experiment with grass-clover leys. Mineral fertilizer was applied alone or combined with a basic application of cattle slurry [1].

		Spring		After 1st cut		After 2nd cut	After 3rd cut
	Annual N rate	Slurry	Mineral	Slurry	Mineral	Mineral	Mineral
	
	kg inorganic N ha^−1^						
Control	0						

Mineral N	60		60				
	120		80		40		
	240		120		80	40	
	360		150		120	60	30
	480		150		120	120	90

Slurry + mineral N	120	60		60			
	240	60	60	60	20	40	
	360	60	90	60	60	60	30
	480	60	90	60	60	120	90

Table 5 gives an overview of the field operations from seeding of the ley under a winter or spring cereals till the last cut of the temporary ley. The operations were carried out with the farmer machineries. The slurry was surface-applied with a 12 m wide trailing hose farm-scale equipment. Therefore, the plots receiving slurry were placed side by side, but in a randomized order.

**Table 5:** Overview of the main field operations.

	Dates	
Operation	SW	MW
*2016*		
Ley seeding with winter wheat	–	August

*2017*		

N fertilization	Spring (30 Mg cattle slurry ha^−1^ and 40 kg mineral N ha^−1^)	Spring (100 kg mineral N ha^−1^)
Ley seeding with spring barley	20-Apr	–
Harvest of cereal crop	July	June
N fertilization prior cuts	July (15 Mg cattle slurry ha^−1^)	June to September (150 kg mineral N ha^−1^ as cattle slurry)
Cut of the ley	September (1 cut)	July to October (4 cuts)

*2018*		

First N fertilization	20-Apr (Mineral N treatments)	4-Apr (Mineral N treatments)
	23-Apr (Slurry + mineral N treatments)	13-Apr (Slurry + mineral N treatments)
First cut	22-May	21-May
Second N fertilization	31-May (Mineral N treatments)	28-May
	2-June (Slurry + mineral N treatments)	
Second cut	19-Jun	26-Jun
Third N fertilization	22-Jun	4-Jul
Third cut	30-Jul	30-Jul
Fourth N fertilization	9-Aug	1-Aug
Fourth cut	6-Sep	3-Sep
Fifth cut	10-Oct	15-Oct

*2019*		

First N fertilization	27-Mar (Mineral N treatments)	30-Mar (Mineral N treatments)
	21-Mar (Slurry + mineral N treatments)	29-Mar (Slurry + mineral N treatments)
First cut	20-May	21-May
Second N fertilization	21-May	23-May (Mineral N treatments)
		30-May (Slurry + mineral N treatments)
Second cut	18-Jun	27-Jun
Third N fertilization	20-Jun	3-Jul
Third cut	30-Jul	29-Jul
Fourth N fertilization	5-Aug	6-Aug
Fourth cut	6-Sep	18-Sep
Fifth cut	10-Oct	23-Oct

The excel file Dataset N fertilization grass-clover 2018-2019.xlsx includes two sheets. The sheet “Dataset” provides the raw data obtained for each treatments, cuts, sites and years. The treatments were replicated four times. The measurements includes botanical composition, %Ndfa, %N and total dry matter. Based on those data, we determined the specific dry matters for each species present, the specific N yields and the clover biological N fixation. However, few data were missing due to technical problems (total dry matter) or lack of biomass to be analyzed (%Ndfa and %N). When necessary for annual calculation (sum of the cuts), those data were estimated by using the average of the other replicates. The sheet “overview correction” presents all corrections that were made. In 2019 at MW site, one replicate of the control treatment and one replicate of the 60 kg mineral N ha^−1^ treatment were discarded due to error in fertilizing the plots.

## 2 Experimental Design, Materials and Methods

### 2.1 Experimental sites and climatic conditions

Two field experiments were conducted on sandy soil in SW site and sandy loam soil in MW site, Denmark (Table 1). At SW site, the ley was composed of 10% white clover (*Trifolium repens L*.) and 90% of different varieties of perennial ryegrass (*Lolium perenne L*.), and was sown with spring barley in April at a density of 24 kg ha^−1^ (Table 5). At MW site, the ley seed mixture consisted of a mixture of 11% red clover (*Trifolium pratense L*.), 7% white clover, 37% perennial ryegrass and 45% festulolium (*Festulolium braunii,* K.A.) that was sown with winter wheat in August at a density of 28 kg ha^−1^. The leys were cut several times in the year of establishment. The biomass was removed after each cut. The climate is temperate and humid (Table 2).

### 2.2 Experimental design and fertilizer treatments

Plots (12 × 3 m) were laid out in the following spring in a randomized plot design with four replicates. Cattle slurry (composition given in Table 3) was applied in half of the plots at a rate of 60 kg available-N ha^−1^ with a trailing hose. Mineral fertilizers, in form of NS 27–4 (13.5% ammonium–N, 13.5% nitrate–N), was either applied alone in the other half of the plots or in combination with the slurry. The rates and timing of application are indicated in Tables 4 and 5. The experiment was repeated in the following year on the same plots. In addition to the N fertilizer, all plots received basic fertilization in both springs with 800 kg ha^−1^ PK 0–4–21 fertilizer (equivalent to 32 kg P ha^−1^, 168 kg K ha^−1^, 53 kg S ha^−1^, 10 kg Mg ha^−1^) and 150 kg ha^−1^ K50 fertilizer (potassium chloride, equivalent to an additional 75 kg K ha^−1^).

The N_2_ fixation activity of clovers in the ley mixtures was estimated using the ^15^N isotope dilution method [2,3]. In the first spring, 1 m^2^ sub-plots (one per plot) were irrigated with ^15^N-(NH_4_)_2_SO_4_ (98 atom%) at a rate corresponding to 0.9 kg N ha^−1^. These sub-plots were used for N_2_ fixation estimation and determination of ley botanical composition in the two growing seasons.

### 2.3 Harvest and biomass analyses

The middle row of 1.5 m width in each plot (12 × 1.5 m) was cut 5 times per year with a grass harvester (Haldrup, Denmark). Prior to harvest of the main plots, 0.25 m^2^ of each sub-plots were manually cut within the ^15^N-labelled plots. The cutting height of the stubble were 5–7 cm in both main and sub-plots.

The total weight of the biomass for each main plot was measured directly on the harvester. The biomass collected in the sub-plots were hand sorted into red clover, white clover, grass (including ryegrass and festulolium fractions), and weed, dried at 60 °C for 48 h and weighed to determine the botanical composition. The fraction of grass, white clover, red clover, total clover, weed are indicated in the column H, I, J, K, L, respectively, of the excel file. The dried plant materials were ball-milled and analyzed for total N and ^15^N using a PDZ Europa ANCA-GSL elemental analyzer interfaced to a PDZ Europa 20-20 isotope ratio mass Spectrometer (Sercon Ltd., Cheshire, UK) and the Dumas dry-combustion method.

### 2.4 Calculations

The percentage of N derived from the atmosphere (%Ndfa) was calculated using grass as reference in the equation of Kristensen et al. [1]:

(1) $$\%Ndfa\;=\;\left(1-\frac{atom\%\;\;{}^{15}N\;\;exces\;in\;clover}{atom\%\;\;{}^{15}N\;excess\;in\;grass}\right)$$

where clover (red or white) and grass atom% excess are calculated by subtracting the atom% ^15^N of clover or grass in unlabeled plots from the atom% ^15^N of clover or grass in the labeled sub-plots. The %Ndfa of white clover - %Ndfa(wc) – and %Ndfa of red clover - %Ndfa(rc) – are indicated in the columns M and N, respectively, of the excel file.

The concentration of N in the grass (N%gr), white clover (N%wc) and red clover (N%rc) are indicated in the columns O, P, Q, respectively, in the excel file.

The dry matter (DM) of the total biomass (column R in the excel file) was obtained by multiplying the total weight (harvester machine) with the percentage of dry weight. The dry matters of grass (column S), white clover (column T), red clover (column U), total clover (column V) and weed (column W) were obtained by multiplying the total dry matter with the fraction of each biomass.

The N yields (NY, expressed in kg N ha^−1^) were calculated by multiplying the dry matter with the N concentration of the respective fraction. The NY of grass, white clover, red clover and total are indicated in columns X, Y, Z, AA, respectively, in the excel file.

The N input via biological N_2_ fixation (qBNF, expressed in kg N ha^−1^) was calculated by multiplying the %Ndfa and N yield of the white clover (wc qBNF, column AB) and red clover (rc qBNF, column AC).

## CRediT authorship contribution statement

**Doline Fontaine:** Formal analysis, Data curation, Writing – original draft, Writing – review & editing. **Rebekka Kjeldgaard Kristensen:** Conceptualization, Methodology, Data curation. **Jim Rasmussen:** Conceptualization, Methodology, Data curation, Writing – review & editing, Supervision. **Jørgen Eriksen:** Conceptualization, Methodology, Writing – review & editing, Supervision, Project administration, Funding acquisition.

## Declaration of Competing Interest

The authors declare that they have no known competing financial interests or personal relationships which have or could be perceived to have influenced the work reported in this article.

## Appendix Supplementary materials

Image, application 1
